# Neural entrainment to rhythmic speech in children with developmental dyslexia

**DOI:** 10.3389/fnhum.2013.00777

**Published:** 2013-11-27

**Authors:** Alan J. Power, Natasha Mead, Lisa Barnes, Usha Goswami

**Affiliations:** Department of Psychology, Centre for Neuroscience in Education, University of CambridgeCambridgeshire, UK

**Keywords:** neural entrainment, developmental dyslexia, low frequency oscillations, temporal sampling, audio-visual

## Abstract

A rhythmic paradigm based on repetition of the syllable “ba” was used to study auditory, visual, and audio-visual oscillatory entrainment to speech in children with and without dyslexia using EEG. Children pressed a button whenever they identified a delay in the isochronous stimulus delivery (500 ms; 2 Hz delta band rate). Response power, strength of entrainment and preferred phase of entrainment in the delta and theta frequency bands were compared between groups. The quality of stimulus representation was also measured using cross-correlation of the stimulus envelope with the neural response. The data showed a significant group difference in the preferred phase of entrainment in the delta band in response to the auditory and audio-visual stimulus streams. A different preferred phase has significant implications for the quality of speech information that is encoded neurally, as it implies enhanced neuronal processing (phase alignment) at less informative temporal points in the incoming signal. Consistent with this possibility, the cross-correlogram analysis revealed superior stimulus representation by the control children, who showed a trend for larger peak *r*-values and significantly later lags in peak *r*-values compared to participants with dyslexia. Significant relationships between both peak *r*-values and peak lags were found with behavioral measures of reading. The data indicate that the auditory temporal reference frame for speech processing is atypical in developmental dyslexia, with low frequency (delta) oscillations entraining to a different phase of the rhythmic syllabic input. This would affect the quality of encoding of speech, and could underlie the cognitive impairments in phonological representation that are the behavioral hallmark of this developmental disorder across languages.

## Introduction

Temporal coding is a critical aspect of speech processing and is fundamental to phonological representation, the mental representation of the sound structure of human languages. Temporal coding is thought to be accomplished in part by the synchronous activity of networks of neurons in auditory cortex that align their endogenous oscillations at different preferred rates with matching temporal information in the acoustic speech signal (Poeppel, 2003; Lakatos et al., 2008; Giraud and Poeppel, 2012). Speech involves auditory, visual and motor modalities, and both auditory and visual information in speech unfold over multiple timescales. Accordingly, oscillating networks of neurons in auditory *and visual* cortices are thought to “phase lock” or “phase align” their ongoing activity with matching modulation rates in the input (Luo et al., 2010). For human speech, the visuo-spatial information generated by face, cheek and mouth movements is temporally predictive of the production of speech sounds, and may “reset” auditory cortex to the optimal phase for processing succeeding vocalizations (Schroeder et al., 2008). *Multi-time resolution models* (MTRMs) of speech processing capitalize on these neurophysiological processes (e.g., Poeppel, 2003; Ghitza and Greenberg, 2009), and argue that the neural entrainment of these oscillatory networks is occurring at multiple temporal rates in both visual and auditory cortices, with hierarchical and interdependent cross-modal phase interactions, resulting in a coherent representation of the signal and enabling communication between human listeners.

A large literature suggests that temporal coding in both the auditory and visual modalities may be atypical in individuals with developmental dyslexia, a specific learning difficulty affecting reading and spelling that affects approximately 7% of children across languages (e.g., Witton et al., 1998; Snowling et al., 2000; Ziegler and Goswami, 2005; Lallier et al., 2009; Facoetti et al., 2010; Goswami et al., 2011; Hämäläinen et al., 2012a). Developmental dyslexia is not due to low intelligence, poor educational opportunities, or overt sensory or neurological damage. The primary cognitive difficulty found in dyslexia across languages is a difficulty in the accurate neural representation of phonology, the sound structure of words. Children with dyslexia are poorer than age- and reading-level matched controls at identifying and manipulating phonological units in words, for example, they are poorer at counting *syllables* (e.g., 3 syllables in “popsicle”), at identifying *rhymes* (e.g., “cat” and “hat” rhyme, “cat” and “hot” do not rhyme), and at recognizing shared *phonemes* (the smallest speech sounds that change meaning, e.g., “clip” and “quip” share the initial phoneme,/k/; see Ziegler and Goswami, 2005, for review). Children with dyslexia are also significantly impaired compared to younger reading level controls in prosodic awareness tasks, such as tasks requiring the identification of syllable stress (Goswami et al., 2013). These difficulties with phonology appear to precede learning to read (Lyytinen et al., 2001), and are also found in children with dyslexia who are learning non-alphabetic scripts. For example, Japanese Kana uses orthographic characters that represent syllables rather than phonemes, and Japanese children with dyslexia find syllable reversal tasks difficult (Kobayashi et al., 2003). Given the importance of neuronal oscillations for speech processing as revealed by multi-time resolution models, it is plausible that the phonological deficits found in dyslexia across languages could be related to impaired or atypical oscillatory mechanisms at one or more temporal rates in either auditory cortex, visual cortex or during audio-visual integration.

Accordingly, and building on the prior work noted above on MTRMs for speech processing, a “temporal sampling” framework (TSF) for developmental dyslexia has been proposed. The TSF suggests that the phonological deficit found in dyslexia across languages might be due in part to impaired or functionally atypical entrainment mechanisms for phonology in auditory cortex, particularly oscillations at the slower temporal rates (theta and delta) that are relevant to syllabic and prosodic processing (Goswami, 2011). As syllable awareness in children develops before phonological awareness of rhymes and phonemes (Ziegler and Goswami, 2005), and as syllables are the primary processing unit in all human languages (Greenberg et al., 2003), atypical entrainment mechanisms related to syllabic phonology would have effects throughout the phonological system in all languages, consequently affecting the phonological representation of smaller units such as rhymes and phonemes. According to multi-time resolution models of speech processing (Giraud and Poeppel, 2012), identification of phonetic segments is related to faster temporal modulations (gamma rate, 30–80 Hz), identification of syllables is related to slower modulations at the theta rate (4–10 Hz), and information relating to syllable stress and prosodic patterning is related to modulations at the delta rate (1.5–4 Hz). Here we provide the first direct test of the TSF with children with developmental dyslexia, utilizing a rhythmic speech paradigm previously developed for typically-developing children (Power et al., 2012b) to measure oscillatory entrainment to phonological information in dyslexia.

Oscillatory entrainment in humans has so far been measured by EEG in rhythmic paradigms, as by hypothesis endogenous oscillations should phase-reset their activity to the rhythmic information in the input, synchronizing cell activity so that peaks in excitation co-occur with stimulus delivery, thereby enhancing neural processing (Lakatos et al., 2005; Canolty et al., 2006). Whereas early studies of oscillatory entrainment in EEG utilized rhythmic streams of non-speech stimuli, such as tones or flashes of light (Lakatos et al., 2008; Stefanics et al., 2010; Gomez-Ramirez et al., 2011), we (Power et al., 2012b) designed a *speech* paradigm based on rhythmic repetition of the syllable “ba” by a female speaker. The repetition rate was 2 Hz, and participating 13-year-old children either saw a “talking head” so that both visual and auditory information was present (audio-visual or AV condition), saw the talking head without sound, so that only visual information was present (visual [V] condition), or heard the stimulus stream in the absence of visual stimulation (auditory [A] condition). The children were asked to detect occasional rhythmic violations in each condition (A, V, AV), when the syllable was slightly late and therefore out of time. We found significant entrainment at the stimulation rate (delta, 2 Hz) in all conditions, and also significant entrainment at the *theta* rate in the auditory and AV conditions. Consistent with the predictions of MTRMs of speech processing, therefore, theta entrainment was important in processing this syllabic input. Furthermore, individual differences in the strength of theta entrainment (measured by inter-trial coherence or phase consistency) were related to measures of phonological processing and reading in this typically-developing child sample. Higher phase consistency was associated with higher behavioral performance. Further, the preferred phase of auditory entrainment was altered by congruent visual information (AV condition), suggesting that visual speech information modulated auditory oscillations to the optimal phase for speech processing in these 13-year-old participants, consistent with Schroeder et al. (2008).

The TSF proposes that auditory oscillatory entrainment to phonological information at both delta and theta rates may by atypical in developmental dyslexia, and that atypical auditory entrainment might also have consequences for visual oscillatory entrainment to speech via cross-modal and cross-frequency phase alignment. The rhythmic speech paradigm that we developed (Power et al., 2012b) can also be used to study entrainment in children with dyslexia. Accordingly, we recruited a group of children with dyslexia, and matched their performance as a group to that of a sub-set of the typically-developing children who had participated in our previous study. The TSF enables a number of plausible predictions with respect to our dyslexic group. The simplest possibility is that the children with dyslexia should show significantly less entrainment to the auditory stimulus stream, at both delta and theta rates (reduced inter-trial coherence or phase consistency). Once cross-modal information is available, however, it is plausible that children with dyslexia may show strength of entrainment that is *equivalent* to typically-developing children (as visual information may modulate auditory oscillations to the optimal phase for speech processing). Indeed, children with dyslexia may rely *more* on visual speech information than typically-developing children, in order to *compensate* for their impaired auditory processing skills. A recent study of audio-visual processing of noise vocoded speech by adults with and without dyslexia produced some evidence for atypical visual processing of low frequency modulations in those with dyslexia in a non-rhythmic paradigm (Megnin-Viggars and Goswami, 2013). Nevertheless, the same study also produced some data suggestive of visual compensation. Other studies of rhythmic entrainment in adults with dyslexia have focused on the auditory modality.

In one relevant study utilizing MEG, we (Hämäläinen et al., 2012b) played amplitude-modulated white noise at 4 temporal rates (2, 4, 10, 20 Hz) to adults with and without dyslexia in an unattended listening paradigm (the participants were watching a silent video). On the basis of the TSF, we expected group differences in neuronal oscillatory entrainment at the slower AM rates (2 Hz, 4 Hz). The data showed significantly less entrainment by the participants with dyslexia in right hemisphere auditory networks to the 2 Hz rate only. There was also significantly weaker entrainment overall (adding across modulation rates) in the right hemisphere for those with dyslexia. As the right hemisphere is thought to prefer slower temporal rates (delta, theta, see Poeppel et al., 2008), these results were considered to be consistent with the TSF. Hamalainen et al. also found that the dyslexic group also showed significantly stronger entrainment to the 10 Hz rate in the *left* hemisphere, a finding which was not predicted. This could indicate compensatory entrainment at faster temporal rates. In a second study investigating dyslexia using EEG and an attended paradigm, we (Soltesz et al., 2013) compared rhythmic entrainment in adults with and without dyslexia to a tone stream delivered at 2 Hz (Soltesz et al., 2013). The task was to press a button whenever white noise replaced a tone in the stream, as in a standard auditory oddball paradigm. In this study, the strength of entrainment as measured by inter-trial coherence (ITC) was significantly reduced in the participants with dyslexia, even though they were as fast and as accurate as the controls in the button-press paradigm. Whereas response time in controls was significantly related to the instantaneous phase of the delta oscillation, with faster responses in the rising phase of the oscillation, participants with dyslexia showed no such relationship. This suggests that the oscillatory function of low frequency brain rhythms may be atypical in dyslexia (Soltesz et al., 2013).

However, an alternative oscillatory framework for dyslexia has been developed by Giraud and her colleagues, who have proposed that a single auditory anomaly, phonemic sampling in left auditory cortex, accounts for the three major aspects of impaired phonological processing in dyslexia (which are impaired phonological awareness, impaired rapid automatized naming [RAN], and impaired phonological memory, see Lehongre et al., 2011; Giraud and Poeppel, 2012). In a passive listening study with adults with dyslexia using MEG, Lehongre et al. (2011) presented amplitude-modulated white noise at rates that increased incrementally from 10 to 80 Hz, and measured the auditory steady state response (ASSR) while participants watched a silent video. Of particular theoretical interest were oscillations in the low gamma band (25–35 Hz), thought to reflect optimal phonemic encoding. Both dyslexic and control participants showed significant phase locking as measured by the ASSR, but hemispheric differences were found between groups, with left-dominant entrainment shown by the control participants only. When faster temporal rates were considered (>50 Hz), then those with dyslexia showed stronger entrainment bilaterally than controls. Lehongre and colleagues then computed the degree of leftward asymmetry shown by each participant at the low gamma rate for ASSR power, and correlated this measure with the phonological measures. Significant relations with phonological processing (a global construct measure made up of Spoonerisms, digit span and non-word repetition) and rapid naming were found when the dyslexics were considered alone, but not for controls alone nor for the total sample. Lehongre et al. (2011) argued that their data suggested a focal (left-lateralized) impairment of selective extraction and encoding of phonemic information, which would not be expected to affect global sensitivity to amplitude modulation. Phonemic oversampling was also proposed by Giraud and Poeppel (2012) to underpin the phonological “deficit” in dyslexia. The oscillatory nesting observed between theta/delta phase and gamma power (Schroeder and Lakatos, 2009; Canolty and Knight, 2010) was argued by Lehongre et al. (2011) to provide a means by which information at the phonemic (gamma) rate is integrated at the syllabic rate.

In the only neuroimaging study of which we are aware to *compare* slow rate (<10 Hz) and faster rate (20 Hz) oscillatory entrainment in dyslexia, the auditory steady state response was recorded to speech-weighted noise stimuli amplitude modulated at either 4 Hz, 20 Hz or 80 Hz (Poelmans et al., 2012). Participants were dyslexic and control adults, the task was passive listening, and EEG recordings were analyzed at parietal and mastoid electrodes only. No group differences were found for the ASSRs to the 80 Hz and 4 Hz stimuli, but a significant group × laterality effect was found for the 20 Hz stimulus. For 20 Hz AM noise, dyslexic adults showed less power at left hemisphere electrodes compared to controls. Phase coherence *between and within hemispheres* was also computed, and a main effect of group was found at the 20 Hz rate for both measures. Adults with dyslexia demonstrated lower inter- and intra-hemispheric coherence than controls. Note that this phase measure is not related to the stimulus *per se*, rather the between-hemisphere results show that the relationship between the phase pattern at the selected electrodes is less similar for participants with dyslexia. As the 20 Hz rate yielded the only significant group differences, Poelmans et al. (2012) concluded that cortical processing of phoneme-rate modulations was impaired in dyslexia.

However, a series of studies with dyslexic adults based on nursery rhymes (rhythmically-produced speech) by Leong and Goswami (2013) has compared rhythmic entrainment in dyslexia at slower and faster rates using behavioral measures (tapping or speaking to a beat). Using modeling developed by Leong (2012), these nursery rhyme studies explored the role of *phase relations* between amplitude modulation at different rates in the speech signal in the perception and production of rhythmic speech. Building on MTRMs of speech processing and the oscillatory hierarchy (Poeppel, 2003; Schroeder et al., 2008; Giraud and Poeppel, 2012), Leong (2012) modeled entrainment to different AM rates in the speech signal using an *amplitude modulation phase hierarchy* (AMPH) approach. Leong assumed that the modulation hierarchy within the speech signal followed the oscillatory hierarchy, with the slowest rates *highest* in the hierarchy. In Leong's models, the slower rates (delta and theta) hence temporally constrain entrainment at the faster rates, such as gamma (for detail regarding these novel AMPH models of the speech signal, see Leong, 2012). Leong and Goswami (2013) demonstrated that participants used the *phase relationship* between delta- and theta-rate AMs (2 Hz and 4 Hz AM rates) to calibrate their rhythmic behavior. Importantly, Leong and Goswami found that adults with dyslexia showed an *earlier* preferred phase angle for theta entrainment compared to control participants. Individual differences in both theta and delta preferred AM phase were correlated with phonological awareness in a Spoonerisms task, and with reading development.

Concerning rhythmic speech *production* (measured by asking participants to speak rhythmically in time with a metronome beat at 2 Hz), Leong and Goswami (2013) reported that the two groups showed equivalent *strength* of entrainment in terms of internal phase locking between delta- and theta-AMs, and between theta- and gamma-AMs (stressed syllable, syllable and phoneme rates, respectively). However, the participants with dyslexia again preferred a different phase alignment of the AMs conveying syllable and phoneme information, respectively (theta- and gamma-AMs). A difference in phase locking *angle* implies a difference in how speech information at different temporal rates is bound together in the final speech percept (Poeppel, 2003). Leong and Goswami (2013) argued that the significant difference in phase-locking angle between syllable- and phoneme-relevant information in speech was consistent with the large behavioral database indicating that phonological information is represented *differently* in the dyslexic mental lexicon.

In the current study, participants are also perceiving rhythmic speech in an attended paradigm, and neuronal oscillatory entrainment can be measured *directly* at both the delta and theta rates of AM (whereas when tapping and speaking in time are the dependent measures, the measurement of entrainment is necessarily indirect because of additional motor demands). Given the preferred phase angle differences found in the studies with adults (Leong and Goswami, 2013), it is therefore possible that the preferred *phase of entrainment* will differ between dyslexic and control children in the current study, at least in the auditory condition, for either delta or theta phase (or both). The phase of entrainment of neuronal oscillation relative to a presented stimulus has been shown to be central to stimulus processing. It has been shown that oscillations entrain to stimuli at differing preferred phases (anti-phase, in fact) depending on whether they are being attended to or being ignored (Lakatos et al., 2008; Besle et al., 2011; Horton et al., 2013). Furthermore, the phase of pre-stimulus delta activity has been shown to be related to reaction times in a task where the target probability was manipulated, suggesting that efficiency of stimulus processing is related to oscillatory phase (Stefanics et al., 2010). EEG phase patterns have also been shown to reflect the selectivity of neural firing with single neurons more likely to fire at specific phases in response to an auditory stimulus (Ng et al., 2013). *These studies suggest that there is an optimal or preferred phase of entrainment which is necessary for accurate and efficient stimulus processing.* If preferred delta and/or theta phase is different for participants with dyslexia, then speech units such as syllables will occur at a sub-optimal phase, and will not be processed optimally. The result will be a degraded representation or encoding of the speech stimulus.

In order to see whether such potential differences in preferred phase would be related to the quality of children's phonological representations, two strategies were employed. First, a phoneme deletion task was administered to participants as a measure of phonological awareness, and was correlated with the entrainment measures. Secondly, a correlogram approach was used to measure the fidelity of the neuronal representation to the envelope information in the speech signal. The speech stimulus is a stream of syllables repeated rhythmically enabling the stimulus envelope to be cross-correlated with the envelope of the averaged neural response. The peak *r*-value from the cross-correlogram gives us an estimate of the strength of stimulus representation in the EEG. The lag at which this peak occurs gives a measure of the timing of stimulus envelope processing (this is a similar approach to Abrams et al., 2009). If the brain is representing a speech syllable with high fidelity but at a different temporal phase with respect to entrainment to the ongoing stimulus, group differences in peak lag would occur, which would again have implications for the overall quality of the phonological representation via the binding together of temporal information at different rates in the speech signal.

To summarize, phase values (entrainment strength or ITC and phase angle), peak *r*-values (correlation strength), and peak lag values (temporal phase measure) might be expected to differ between dyslexic and control participants at delta and theta rates according to the TSF. According to the model based on anomalous temporal sampling at the low gamma rate proposed by Lehongre and colleagues, no such differences might be expected. In contrast, it has also been proposed that dyslexic children are developing high-quality mental representations of speech, and that the cognitive “phonological deficit” found in dyslexia arises as a result of problems in *accessing* the mental lexicon (see Ramus and Szenkovits, 2008). If the neural phonological representations themselves are precise, then on this “intact representations” hypothesis no group differences in these neural measures of representational quality would be expected.

## Materials and methods

### Participants

We studied 21 typically-developing children and 11 children with a history of developmental dyslexia (mean ages of 165.57 ± 12.71 months and 166.73 ± 13.72 months, respectively). All children were taking part in a longitudinal behavioral study of auditory processing (Goswami et al., 2011). All participants and their guardians gave informed consent for EEG in accordance with the Declaration of Helsinki, and the study was approved by the Psychology Research Ethics Committee of the University of Cambridge. All participants were free of any diagnosed learning difficulties aside from dyslexia (i.e., dyspraxia, ADHD, autistic spectrum disorder, speech and language impairments) and spoke English as their first language.

### Standardized tests of reading, nonword reading, vocabulary and IQ

Psychometric tests were given for the purposes of group matching and also exploring possible relations between entrainment and the development of spoken and written language skills. The psychometric tests comprised the British Ability Scales (BAS) (single word reading, Elliott et al., 1996); the single word reading (SWE) and phonemic decoding efficiency (PDE) measure of non-word reading from the TOWRE (Torgesen et al., 1999); the British Picture Vocabulary Scales (BPVS receptive vocabulary, Dunn et al., 1982); and one subtest of the Wechsler Intelligence Scale for Children (WISC-III, Wechsler, 1992): picture arrangement. Performance on these measures is shown in Table 1.

**Table 1:** **Group differences in Age, IQ, and behavior**.

**Measure**	**CA**	**DY**	***F*_(1, 30)_**	***p***
Age (months)	165.57 ± 12.71	166.73 ± 13.72	0.057	0.814
IQ	112.76 ± 13.31	114.64 ± 14.07	0.138	0.713
BAS standard score	109.29 ± 11.86	86.18 ± 15.5	22.729	<0.001
Reading age (months)	177.00 ± 20.7	134.55 ± 27.52	24.186	<0.001
TOWRE word reading	103.48 ± 10.33	87.91 ± 7.82	19.125	<0.001
TOWRE non-word reading	107.62 ± 11.21	81.55 ± 10.99	39.559	<0.001
BPVS not aligned	107.71 ± 13.28	100.1 ± 19.48	1.604	0.215
RAN TOTAL	34.67 ± 3.95	38.91 ± 8.88	3.5830	0.070
pSTM	45.81 ± 11.25	35.91 ± 12.55	5.170	0.03
Phoneme deletion	16.48 ± 3.17	12.91 ± 3.89	7.825	0.009

### Experimental phonological tasks

In order to see whether individual differences in entrainment would relate to individual differences in phonological processing between children, participants were administered a phoneme deletion task, an experimental measure of phonological short-term memory (PSTM) and an experimental measure of rapid automatized naming (RAN). Further details for each task are given in Power et al. (2012b).

### Rhythmic entrainment task

**Figure 1:**
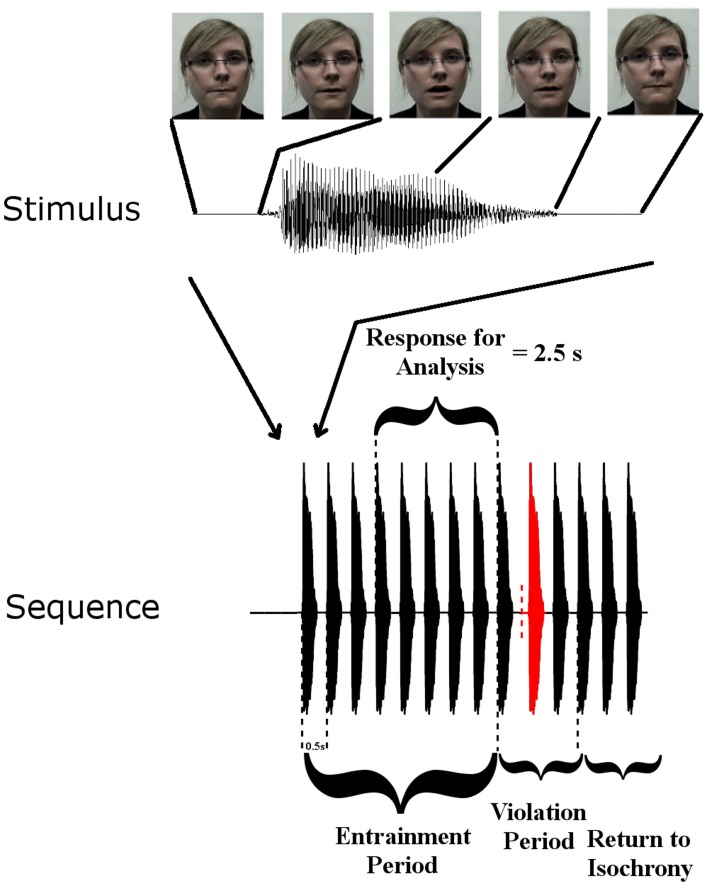
**Stimulus Setup: top panel shows one auditory “Ba” token and corresponding frames of the visual stimulus at five time points**. Visual movement initiates 68 ms before auditory onset. The lower panel shows a stimulus sequence consisting of the entrainment period with SOA of 500 ms, and the violation period where SOA is disrupted, followed by 3 re-entrainment stimuli (“Return to Isochrony”). The red stimulus is the violator, whose position in the violation period is chosen at random (i.e., either the first, second, or third stimulus can violate the rhythm). The vertical dashed red line indicates where the stimulus would have onset if it had adhered to the isochronous stimulation rate. Also shown is the “Response for Analysis” period over which EEG responses were analyzed.

Rhythmic speech comprising multiple repetitions of the syllable “BA” was presented at a uniform repetition rate of 2 Hz. There were three conditions: auditory (A), visual (V), and audio-visual (AV). Further details of the task can be found in Power et al. (2012b). Figure 1 summarizes the paradigm.

### EEG preprocessing

This was exactly as in Power et al. (2012b).

### EEG analysis

**Figure 2:**
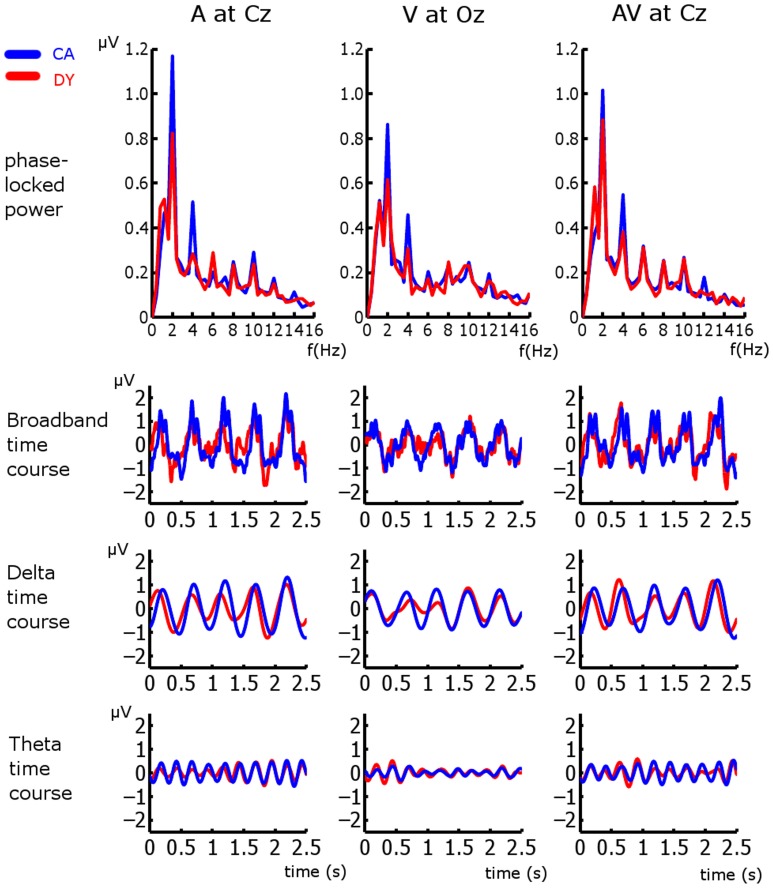
**Frequency spectra and broadband, delta, and theta time courses for all three conditions at representative electrodes**. CA responses are in blue and DY response are in red.

For all analyses, the first three observations in each entrainment period were discarded to ensure that rhythmicity had been established (following the approach employed in Gomez-Ramirez et al., 2011). Here we are interested in entrainment to a uniform stimulus repetition rate, and so responses in the violation and “return to isochrony” periods (see Figure 1) were not analyzed. Furthermore, sequences in which a target was not detected were discarded, as were catch trials. As accuracy was ~79% and 75 target sequences were presented per condition, the analysis included ~60 trials per subject per condition. In order to identify frequency bands of interest we examined the phase-locked power (i.e., the power of sequence averages in the time period of interest) in the three conditions (see Figure 2). Phase locked power was obtained as in Power et al. (2012b). Given the peaks evident in the spectra, with the highest phase-locked power present for delta and theta, we deemed the delta (~2 Hz) and theta (~4 Hz) frequency bands to be of interest (for further details see *Results* and *Discussion*). Frequency band activity was obtained using FIR filters designed using the Parks-McClellan algorithm (Parks and McClellan, 1972). The delta band filter had corner frequencies of 1 and 3 Hz and the theta band filter had corner frequencies of 3 and 5 Hz. Both had a 40 dB attenuation in the stop band. In order to examine whether auditory entrainment differed for the A and AV conditions, we subtracted an estimate of phase-locked visual activity from each AV trial (AV-V), and compared the remaining A and (AV-V) activity. The estimate of visual activity was obtained from the time-locked average activity in the visual condition.

#### Power analysis

We wished to investigate possible differences in overall power between conditions and groups. This is important both in terms of potentially different EEG power in response to the various conditions, but also for interpreting differences in strength of phase locking. Higher inter-trial coherence (ITC) values may only be interpreted as improved phase consistency over trials if they are accompanied by no change in response power. If higher ITC is accompanied by a change in response power, it is possible that this is due to a stronger additive response as opposed to increased consistency over trials. To obtain the overall total power we calculated the FFT of the broadband responses for each trial for each subject and took the average. Thus, both phase-locked and non-phase locked power are included in the measure. Delta and theta power were extracted by taking the power at 2 and 4 Hz, respectively, from the overall broadband frequency representation.

#### Assessing phase-locking

**Figure 3:**
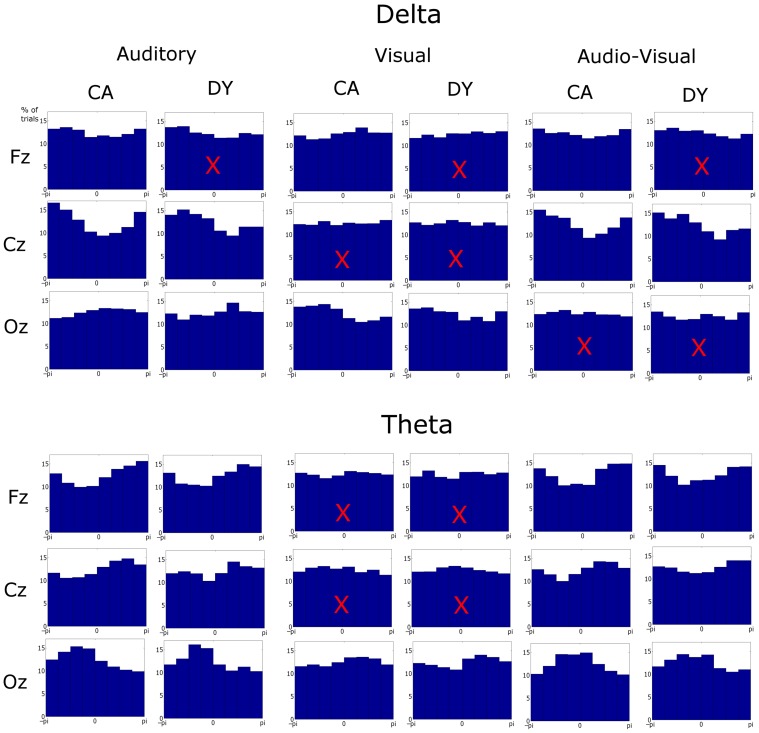
**Phase distributions at stimulus onset at representative frontal, central, and occipital electrodes in each condition, frequency band, and group**. The *x*-axis is phase ranging from −π to π and *y*-axis represents the percentage of trials. Most distributions differed from uniformity when tested against the Rayleigh statistic at a critical *p*-value of 0.001. Distributions with a superimposed X did not result in significant entrainment.

The pre-stimulus phase of the last 5 stimuli in the entrainment period was obtained. The pre-stimulus phase is defined here as the phase at the onset of the visual element of the stimulus in the AV condition. This time point is kept consistent for all conditions (i.e., for the auditory condition phases are extracted at the time point where the visual stimulus would have onset, had the visual element of the stimulus accompanied the auditory information, this is 68 ms before auditory stimulus onset). Only sequences where the rhythmic violation was correctly identified are analyzed. These phase values were pooled across sequences and subjects. Given that 75 target sequences were presented to each subject and accuracy was ~79%, the number of phase observations was 6270, 6355, and 6155 (~60 sequences × 5 stimuli × 21 participants) observations for the control group for the auditory, visual and AV conditions, respectively. Similarly 3185, 3210, and 3250 observations were tested for the dyslexic group in the three conditions, respectively (~60 sequences × 5 stimuli × 11 participants). Pre-stimulus phase distribution histograms for each condition were obtained (see Figure 3). The phase values were extracted by obtaining the *analytic signal* of the filtered responses via the Hilbert transform. The *analytic signal* is complex, i.e., it has real and imaginary components, and thus the instantaneous phase can be extracted. To test if pre-stimulus phase distributions differed from uniformity, the distributions for the three conditions were tested against the null hypothesis of uniformity using the Rayleigh statistic at three representative electrodes (Fz, Cz, and Oz). A critical *p*-value of 0.001 was selected to minimize type I error. Statistical difference from uniformity suggests a preferred concentration of phase values, which is indicative of entrainment (Stefanics et al., 2010; Gomez-Ramirez et al., 2011).

**Figure 4:**
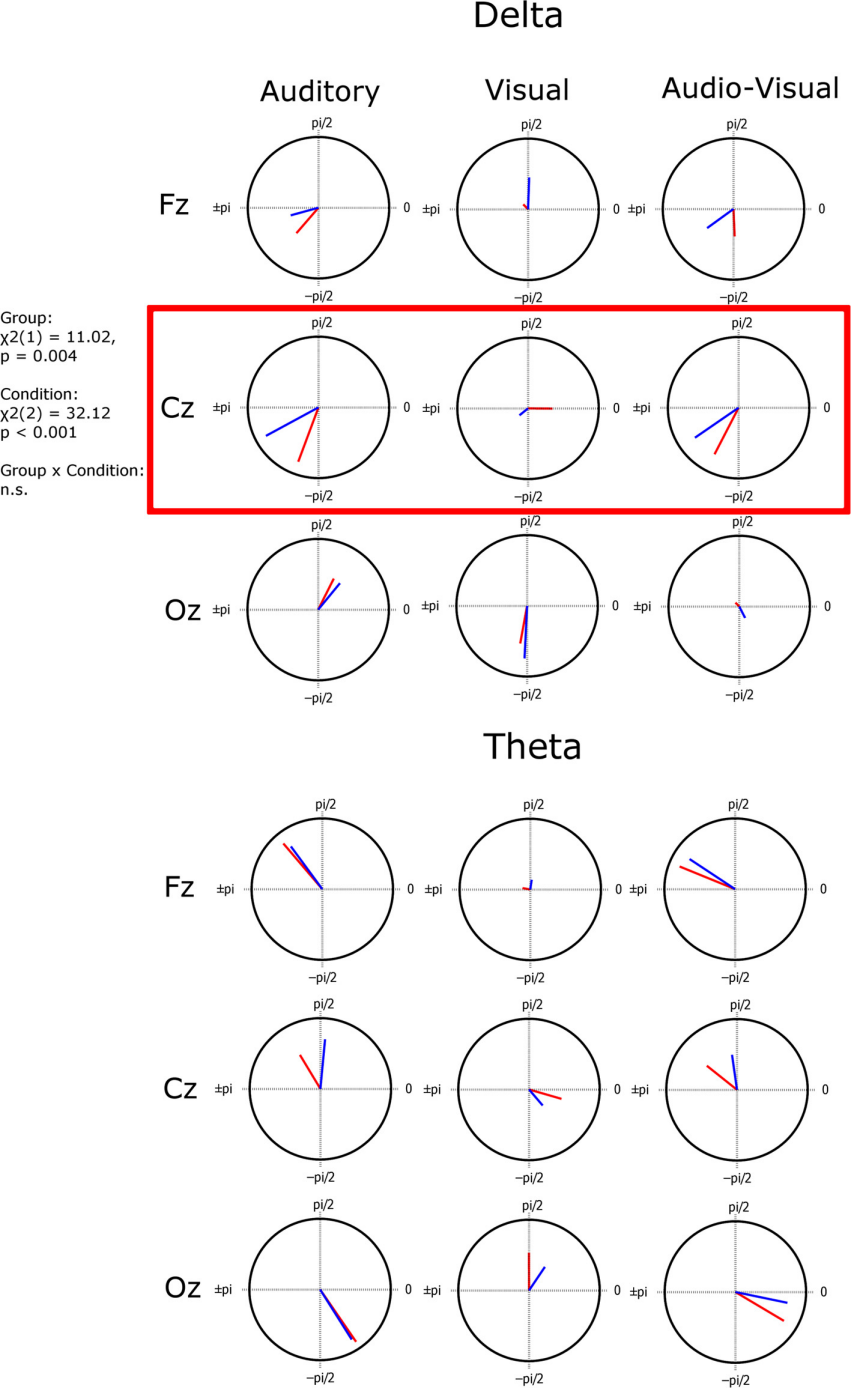
**Mean resultant vector plots indicating the coherency of preferred phase across subjects (the length of the vector) and the preferred angle of entrainment (the vector angle) plotted on a unit circle**. A significant group difference in preferred angle was found in the delta band at electrode Cz.

Inter-trial coherence (ITC) was then used to compared strength of entrainment across groups, conditions, and channels. ITC is a measure of phase alignment and can have values ranging from 0 to 1. 1 indicates perfect phase alignment and 0 indicates no phase alignment. ITC was calculated for the same pre-stimulus phase values that were submitted to the Rayleigh test. Preferred phase of entrainment between groups and conditions was also investigated (shown in Figure 4). The preferred phase of entrainment for each participant is obtained by calculating the mean pre-stimulus phase for that individual. Mean preferred phase for each group is then calculated for each condition (A, V, AV). If the phase at which the low frequency oscillations (delta, theta) entrain is different between the groups, this implies that the information encoded is different (neurons are firing at the “wrong” time, thus selectively encoding information at a sub-optimal point in the stimulus).

#### Cross-correlogram analysis of entrainment and laterality

**Figure 5:**
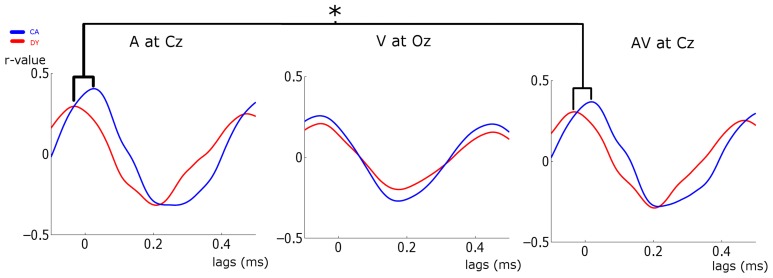
**Stimulus-Response cross-correlation analysis**. The plots show the cross-correlation analysis between the stimulus and the responses at representative electrodes. Average CA data is plotted in blue and DYs in red. A very strong representation of the temporally extended stimulus is seen in the response. Significant peak timing differences are indicated. ^*^p < 0.05.

Finally, in order to obtain converging evidence for entrainment, the relationship between the stimuli and the neural responses was also assessed using cross-correlations (see Figure 5). We then sought to relate measures of stimulus representation in the EEG data, obtained from these cross-correlations, to the behavioral data. To do this we employed peak *r*-values and the lags at which those peaks occurred. Peak *r*-values are a measure of the strength of stimulus representation in the EEG, and peak-lags are a measure of stimulus-response timing. We also tested potential hemispheric differences in the strength and timing of auditory encoding, following Abrams et al., 2009. To do this we found peak *r*-values and the lags at which those peaks occurred at three pairs of temporal electrodes. The temporal electrode pairs were electrodes at (T3, T4), (T5, T6) and (Tp7, Tp8) of the 10–20 system. The first electrode of each pair was in the left temporal region and the second was in the right temporal region.

#### Phase re-setting of auditory oscillatory activity by visual information

Finally, we sought to investigate the impact of the accompanying visual stimulation on auditory entrainment. The pre-stimulus phase values (at auditory stimulus onset) for the AV and (AV-V) responses were extracted in the same manner as outlined above for the separate conditions. We then looked at the topography of the strength of entrainment. To do this we plotted the pooled phase values at each electrode (shown in Figure 7). These topographies show a common region of strong entrainment, indicative of entrainment in auditory areas (see Figure 7). Subsequent analysis was thus confined to the pooled activity of electrodes in this region of interest (ROI). The electrodes chosen for this ROI are shown in Figure 7. We compared the extent of phase alignment as obtained using ITC and the preferred pre-stimulus phase. Once again an estimate of the preferred phase was determined for each subject by finding the mean pre-stimulus phase.

## Results

### Behavioral entrainment task

In order to assess whether there were significant behavioral differences between conditions, 2 Two-Way mixed design ANOVAs with a between-subject factor of Group and a within-subject factor of Condition were carried out. The dependent variables in the separate ANOVAs were the EEG task (79.4% accuracy) behavioral threshold in ms in each condition and response time (RT) in ms in each condition, respectively. If the assumption of sphericity was violated the Greenhouse–Geisser corrected degrees of freedom are reported. The ANOVA for *threshold* showed a main effect of Group that approached significance, *F*_(1, 30)_ = 4.006, *p* < 0.054, η^2^_*p*_ = 0.118. There was a significant main effect of condition, *F*_(1.57, 47.089)_ = 97.9, *p* < 0.001, η^2^_*p*_ = 0.765. *Post-hoc* inspection of the means (Bonferroni corrected) showed that the threshold for the visual condition was significantly higher than the thresholds for the auditory and AV conditions (both *p*'s < 0.001). The thresholds in the auditory and AV conditions did not differ from each other (*p* > 0.05). The Group × Condition interaction approached significance [*F*_(1.57, 47.089)_ = 2.602, *p* = 0.097]. *A priori*, we had expected potential group differences in benefit accrued in presenting AV over A or V alone and also a possible differential benefit by group of A over V (those with dyslexia worse in A and better in V). Therefore, we carried out three planned exploratory *post-hoc t*-tests probing group effects in differences between conditions: (DY_A-DY_AV) vs. (CA_A-CA_AV), (DY_V-DY_A) vs. (CA_V-CA_A), (DY_V-DY_AV) vs. (CA_V-CA_AV). With Bonferroni corrections, a significance threshold of *p* = 0.05/3 = 0.016 was applied. Results of these *post-hoc* tests showed that dyslexics gained significantly more benefit in the AV condition compared to the auditory alone condition (*p* = 0.014). The difference in benefit from visual alone to AV did not differ between groups (*p* = 0.057). Therefore, the *post-hoc t*-tests suggest that dyslexics accrued more benefit than controls when stimuli were presented audio-visually rather than as auditory-alone. The same pattern was not found for AV presentation over visual-alone. The advantage of auditory alone over visual alone presentation was not significantly different between the groups (*p* = 0.802).

The ANOVA for *response time* showed a main effect of condition, [*F*_(1.638, 49.139)_ = 39.24, *p* < 0.001, η^2^_*p*_ = 0.567], but no significant group effects [*F*_(1, 30)_ = 0.035, *p* > 0.05] nor interaction [*F*_(1.638, 49.139)_ = 0.118, *p* > 0.05]. *Post-hoc* inspection of the significant condition effect (Bonferroni corrected) showed that RT in the visual condition was significantly faster than RT for the auditory and AV conditions (both *p*'s < 0.001). Differences in RT between the auditory and AV conditions approached significance (*p* = 0.054), suggesting that although the AV condition did not result in an improved detection threshold over auditory information alone, some facilitation of RT was occurring. Performance on the behavioral entrainment task is shown in Table 2.

**Table 2:** **Response times and 79.4% detection threshold (in ms) for the EEG behavioral task**.

	**CA**	**DY**
RT auditory (ms)	352.44 ± 45.48	358.52 ± 44.31
RT visual (ms)	303.27 ± 48.82	303.76 ± 43.45
RT audio-visual (ms)	337.71 ± 41.29	339.61 ± 40.02
EEG behavioral threshold auditory (ms)	51.39 ± 19.34	80.01 ± 62.15
EEG behavioral threshold visual (ms)	131.21 ± 26.44	138.56 ± 38.69
EEG behavioral threshold audio-visual (ms)	56.00 ± 17.85	62.41 ± 28.68

**Figure 6:**
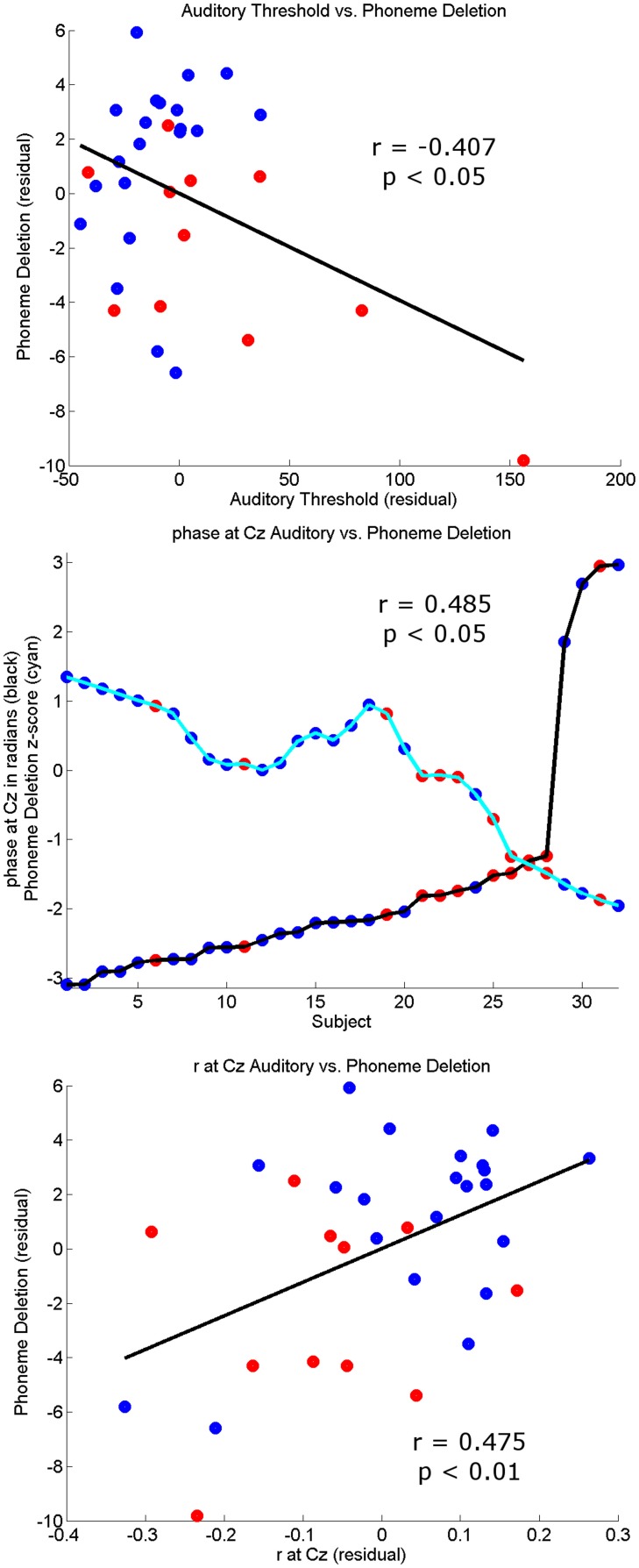
**Correlation plots**. The **top panel** shows the partial correlation plot controlling for age and IQ of the Auditory Threshold on the EEG behavioral task with phonology (phoneme deletion). The **middle panel** shows the relationship between the circular variable preferred phase of entrainment (black trace) and the linear variable phoneme deletion *z*-score (cyan). For visualization purposes the phoneme deletion trace has been smoothed using a 12-point averaging window. Higher phoneme deletion scores occur when a subject has a preferred phase in the region (−π, −π/2) and lower scores in the region (π/2, π). The **bottom panel** shows the partial correlation plot controlling for age and IQ of strength of auditory stimulus representation at Cz (*r*) with phonology (phoneme deletion). Controls subjects are identified by blue dots and dyslexics by red dots in all panels.

Finally, to check that individual differences in the thresholds for the 3 conditions were correlated with the behavioral, reading, and phonological measures, partial correlations across all subjects controlling for age and IQ were computed (see Table 3). Most of the correlations were significant, suggesting that the task is tapping into mechanisms that are relevant to reading and reading development. The top panel of Figure 6 shows a scatter plot and regression line of the auditory threshold in the EEG behavioral task plotted against performance in the phoneme deletion task.

**Table 3:** **Partial correlations across all subjects controlling for age and IQ between EEG behavioral task thresholds and reading and phonology measures**.

**Measure**	**Auditory threshold**	**Visual threshold**	**Audio-visual threshold**
BAS (SS)	*r* = −0.579^**^	*r* = −0.508^**^	*r* = −0.383^*^
Reading age	*r* = −0.522^**^	*r* = −0.475^**^	*r* = −0.305
TOWRE word reading	*r* = −0.434^*^	*r* = −0.441^*^	*r* = −0.306
TOWRE non-word reading	*r* = −0.538^**^	*r* = −0.533^**^	*r* = −0.389^*^
RAN	*r* = 0.193	*r* = 0.265	*r* = 0.216
pSTM	*r* = −0.044	*r* = −0.114	*r* = 0.153
Phoneme deletion	*r* = −0.407^*^	*r* = −0.123	*r* = −0.313

** p < 0.01,

* p < 0.05.

### EEG data: total response power

To assess potential group differences in *total response power*, we carried out separate ANOVAs for each frequency band of interest (delta, theta) with the between-subject factor of group and within-subject factors of condition and channel. For the delta band ANOVA we found no main effect of group [*F*_(1, 30)_ = 0.104, *p* = 0.75, η^2^_*p*_ = 0.003]. There were significant main effects of condition [*F*_(2, 60)_ = 9.932, *p* < 0.001, η^2^_*p*_ = 0.249] and channel [*F*_(2, 60)_ = 10.062, *p* < 0.001, η^2^_*p*_ = 0.251]. The condition effect was driven by larger delta power in the A and AV conditions than the V condition. Power in the A and AV conditions did not differ. The main effect of channel was driven by higher delta power at Fz than at Cz and Oz. There was no difference in delta power between Cz and Oz. The was also a significant group × condition interaction [*F*_(2, 60)_ = 3.428, *p* < 0.039, η^2^_*p*_ = 0.103]. *Post-hoc* tests showed that this was driven by higher delta power in the A than V condition for controls, compared with no difference for those with dyslexia. In contrast, those with dyslexia had higher delta power for the AV condition than the V condition; this was not the case for controls.

In the theta band ANOVA we again found no main effect of group, suggesting that overall theta power was similar between the groups [*F*_(1, 30)_ = 0.233, *p* = 0.633, η^2^_*p*_ = 0.008]. Once again there were significant main effects of condition [*F*_(2, 60)_ = 7.116, *p* = 0.002, η^2^_*p*_ = 0.192] and channel [*F*_(2, 60)_ = 3.875, *p* < 0.026, η^2^_*p*_ = 0.114]. The condition effect was again driven by larger delta power in the A and AV conditions than in the V condition. Power in the A and AV conditions did not differ. The main effect of channel was driven by higher delta power at Fz than at Oz. There was also a significant group × channel interaction [*F*_(2, 60)_ = 4.459, *p* < 0.026, η^2^_*p*_ = 0.129]. *Post-hoc* testing revealed that this was driven by larger theta power at Fz than Cz for DYs only. There was also a significant channel × condition interaction [*F*_(2.974, 89.229)_ = 5.036, *p* = 0.003, η^2^_*p*_ = 0.144]. This is to be expected, as different channels should respond differently to different conditions e.g., Cz would respond more strongly to auditory than visual stimulation.

### EEG data: phase consistency

We next explored entrainment in the pre-stimulus phase distributions of the delta and theta activity (see Figure 3). Here three representative electrodes were chosen for analysis: Fz, Cz, and Oz, identifying responses from frontal, central, and occipital regions, respectively. Significant phase locking (i.e., significant differences from a uniform random distribution) were investigated using the Rayleigh statistic, and a critical *p*-value of 0.001 was chosen in order to minimize Type I errors.

In the *auditory* condition, significant entrainment was found at both delta and theta rates at all three channel locations, with one exception, the Fz channel for dyslexic participants (A_δ__DY_Cz: *Z* = 36.76, *p* << 0.001; A_δ__DY_Oz: *Z* = 7.27, *p* << 0.001; A_δ__CA_Fz: *Z* = 14.30, *p* << 0.001; A_δ__CA_Cz: *Z* = 124.55, *p* << 0.001; A_δ__CA_Oz: *Z* = 36.76, *p* << 0.001; A_θ__DY_Fz: *Z* = 28.43, *p* << 0.001, A_θ__DY_Cz: *Z* = 9.60, *p* << 0.001; A_θ__DY_Oz: *Z* = 35.97, *p* << 0.001; A_θ__CA_Fz: *Z* = 73.58, *p* << 0.001; A_θ__CA_Cz: *Z* = 49.58, *p* << 0.001; A_θ__CA_Oz: *Z* = 75.38, *p* << 0.001). Regarding delta activity at Fz for the dyslexics, the entrainment did approach significance (A_δ__DY_Fz: *Z* = 6.73, *p* = 0.0013). Therefore, as would be expected on MTRMs of speech processing, theta entrainment to the syllable stimulus was present in both groups and at all electrodes. Significant delta entrainment was also present in both groups at all electrodes, as would be expected in our paradigm.

In the *visual* condition, entrainment was significant in occipital areas only, as would be expected (V_δ__DY_Oz: *Z* = 8.81, *p* << 0.001; V_δ__CA_Oz: *Z* = 46.28, *p* << 0.001; V_θ__DY_Oz: *Z* = 10.01, *p* << 0.001; V_θ__CA_Oz: *Z* = 13.30, *p* << 0.001). No significant entrainment was found at Cz in either band (V_δ__DY_Cz: *Z* = 0.56, *p* > 0.05; V_δ__CA_Cz: *Z* = 0.41, *p* > 0.05; V_θ__DY_Cz: *Z* = 3.78, *p* > 0.05; V_θ__CA_Cz: *Z* = 5.22, *p* > 0.05). While significant entrainment was not found in either band at Fz for dyslexics (V_δ__DY_Fz: *Z* = 0.70, *p* > 0.05; V_θ__DY_Fz: *Z* = 0.51, *p* > 0.05), controls did show significant entrainment at Fz in the delta band (V_δ__CA_Fz: *Z* = 13.09, *p* < 0.001) but not the theta band (V_θ__CA_Fz: *Z* = 2.04, *p* > 0.05).

The pattern of entrainment for the *audio-visual* condition was somewhat more complex. Both groups showed significant entrainment in the theta band at Fz, Cz and Oz (AV_θ__DY_Fz: *Z* = 21.10, *p* << 0.001, AV_θ__CA_Fz: *Z* = 75.50, *p* << 0.001; AV_θ__DY_Cz: *Z* = 9.78, *p* << 0.001; AV_θ__CA_Cz: *Z* = 35.83, *p* < 0.001; AV_θ__DY_Oz: *Z* = 21.03, *p* < 0.001; AV_θ__CA_Oz: *Z* = 66.55, *p* < 0.001). In the delta band, however, both groups showed significant entrainment at Cz only (AV_δ__DY_Cz: *Z* = 34.43, *p* << 0.001; AV_δ__CA_Cz: *Z* = 78.22, *p* < 0.001). Controls also showed significant entrainment at Fz (AV_δ__CA_Fz: *Z* = 9.50, *p* << 0.001), whereas for the dyslexics entrainment only approached significance at Fz (AV_δ__DY_Fz: *Z* = 6.64, *p* = 0.0013). Neither group showed significant entrainment at Oz (AV_δ__DY_Oz: *Z* = 1.58, *p* > 0.05; AV_δ__CA_Oz: *Z* = 2.11, *p* > 0.05). The Oz data is likely due to volume conduction from auditory areas. As can been seen from Figure 3, activity at Oz in the auditory condition tends to entrain in opposite phase to the visual condition. This would lead to a balancing of the audio-visual phase distribution at Oz.

### EEG data: phase locking strength (ITC)

In order to examine potential group differences in the degree of phase locking *consistency* for each group we carried out separate mixed factor ANOVAs by group for each frequency band of interest. The with-in group factors were condition (A vs. V vs. AV), and channel (Fz vs. Cz Vs. Oz). Once again, if the assumption of sphericity was violated the Greenhouse–Geisser corrected degrees of freedom are reported. In the *delta band* ANOVA there was no main effect of group [*F*_(1, 30)_ = 0.519, *p* = 0.477, η^2^_*p*_ = 0.017], hence the strength of entrainment did not differ between the groups. There was a significant effect of condition [*F*_(2, 60)_ = 8.294, *p* = 0.001, η^2^_*p*_ = 0.217]. Bonferroni corrected *post-hoc* analysis showed this to be driven by stronger entrainment in the auditory and audio-visual conditions than in the visual condition. Strength of entrainment was equivalent between auditory and audio-visual conditions. There was also a main effect of channel [*F*_(2, 60)_ = 14.74, *p* < 0.001, η^2^_*p*_ = 0.329]. *Post-hoc* analysis found this to be driven by stronger entrainment at Cz then at either Fz or Oz. Finally, there was a significant condition × channel interaction [*F*_(4, 120)_ = 9.474, *p* < 0.001, η^2^_*p*_ = 0.240]. This interaction suggests that strength of entrainment at the electrodes depends on the experimental condition. This is to be expected, e.g., we would expect Cz to show stronger entrainment to the auditory and audio-visual stimuli than the visual stimulus. This can be seen in Figure 4. No other significant effects or interactions were found in the delta band.

In the theta band ANOVA the main effect of group approached significance [*F*_(1, 30)_ = 3.264, *p* = 0.081, η^2^_*p*_ = 0.098]. This suggests that the strength of theta entrainment tends to be greater for controls than those with dyslexia. A main effect of condition was also found [*F*_(2, 60)_ = 5.916, *p* = 0.005, η^2^_*p*_ = 0.165]. As in the delta band *post-hoc* analysis (Bonferroni corrected), entrainment in the auditory and audio-visual conditions was significantly stronger than in the visual condition. There was also a main effect of channel [*F*_(1.468, 44.031)_ = 5.576, *p* = 0.013, η^2^_*p*_ = 0.157]. Bonferroni *post-hoc* tests showed this to be driven by stronger entrainment at Oz than Cz, No significant interactions were found (all *p*'s > 0.05).

### EEG data: preferred phase of entrainment

Having assessed both presence of entrainment (significant phase locking, ITC) and potential differences in strength of entrainment for each group (degree of consistency in phase locking), we sought to investigate potential group differences in the *preferred phase* of entrainment. Although consistency of phase (strength of phase locking) did not differ between groups, this does not mean that both groups entrained at the *same* phase. The preferred phase of entrainment has been shown to be a crucial contributor to stimulus processing (Lakatos et al., 2008; Ng et al., 2013). Preferred phase angles can be seen in Figure 4. The length of the vector in Figure 4 represents the inter-subject coherence; the greater the magnitude of the vector, the more similar the phase at which all subjects entrain. Conversely the shorter the vector, the less consistent (or more variable) the phase across subjects. In order to investigate whether preferred phase differed between groups, we carried out 6 group × condition ANOVAs, one for each frequency band/channel combination (Cz and delta, Cz and theta, Fz and delta, Fz and theta, Oz and delta, Oz and theta). This was done using the Harrison-Kanji two-factor ANOVA test (HK ANOVA) for circular variables (Harrison and Kanji, 1988). This test is not carried out using repeated measures. Also, the reported statistic depends on the width, or concentration factor kappa, of the Von Mises distribution applied to the data. If it is low (<2), a Chi-squared statistic is reported, but if it is high, an F-statistic is reported. A significant group effect was found only for Cz in the delta band ANOVA (χ^2^_(1)_ = 11.02, *p* = 0.004). A significant main effect of condition was also found in this ANOVA (χ^2^_(2)_ = 32.12, *p* <0.001). The group × condition interaction was not significant (*p* > 0.05). Since the entrainment analysis and Figure 3 showed that activity at Cz in the visual condition was not significantly entrained, and thus the preferred phase for this condition at this channel is not informative, we carried out a further exploratory group (CA vs. DY) × condition (A vs. AV) HK ANOVA for Cz and delta band activity, leaving out the potentially confounding visual condition. Again we found a significant main effect of group [*F*_(1, 63)_ = 9.08, *p* = 0.0038]. There was, however, no longer an effect of condition (*p* > 0.05) and no significant interaction (*p* > 0.05). This suggests that the two groups differ in their preferred phase of entrainment in the auditory and audio-visual conditions at Cz, and that the preferred phase for each group does not differ between these conditions. Activity at Cz is broadly indicative of auditory processing in this task.

### Summary of EEG data

Regarding our hypotheses about potential group differences in entrainment, these data suggest that there were no overall group differences in response power or in the consistency of phase across trials (ITC). However, there were important group differences in the *preferred phase of entrainment*, which differed at Cz in the delta band in the Auditory and AV conditions. This points toward a potentially very important difference between the groups in the oscillatory processes supporting speech encoding, one that may have significant implications for the quality and type of information that is encoded. In particular, if the different preferred phase of entrainment has a negative effect on speech encoding by children with dyslexia, this should be reflected in relationships between individual differences in preferred phase and the behavioral measures of reading and phonology. To investigate whether this was the case, circular-linear correlations between the preferred phase of delta entrainment and the behavioral measures were computed, and are shown in Table 4. For the auditory condition, significant correlations are shown for all the measures of reading and for phoneme deletion, with a trend toward significance (*p* < 0.10) for the phonological memory and rapid naming measures. Clearly, preferred phase is significantly related to the quality of the phonological representations in the mental lexicons of our participants. A plot outlining the relationship between preferred phase of entrainment at Cz in the auditory condition and performance in the phoneme deletion task can be seen in the middle panel of Figure 6. This important result is considered further in the Discussion.

**Table 4:** **Circular-linear correlation between preferred delta phase of entrainment and reading and phonology measures**.

**Measure**	**Preferred phase for A at Cz**	**Preferred phase for V at Oz**	**Preferred phase for AV at Cz**
BAS(SS)	0.532^*^	0.322	0.322
BAS(AS)	0.570^**^	0.213	0.294
Reading age	0.388^*^	0.245	0.356
TOWRE word reading	0.510^*^	0.200	0.323
TOWRE non-word reading	0.465^*^	0.324	0.271
RAN (combined)	0.405^+^	0.290	0.447^*^
pSTM combined	0.412^+^	0.312	0.424^+^
Phoneme deletion	0.485^*^	0.144	0.389^+^

The pattern of correlations mirrors that of the peak lag correlations in this table emphasizing that both measures tap into similar mechanisms (that is the timing of the EEG activity in response to the stimulus).

** p < 0.01,

* p < 0.05,

+ p < 0.1.

### Quality of the stimulus representation: cross-correlograms

The analyses conducted so far have investigated differences in EEG responses between the groups. Now we investigate the direct relationship between the EEG response and the entraining stimulus for each group. To ascertain this relationship, we calculated the cross-correlogram between the stimulus envelope and the neural response. Following Abrams et al. (2009), we did not partition the EEG into sub-bands for this analysis, but used the broadband response. The peak *r*-values of the cross-correlogram indicate the strength of stimulus envelope representation in the EEG response. The lags at which the peak *r*-value occurs indicate the timing/phase at which the greatest representation of the stimulus occurs. Given the significant differences in preferred phase found in the EEG, peak lag values in particular might be expected to differ between those with dyslexia and the control group.

The strength of stimulus representation was investigated using a mixed factor ANOVA with the between-subject factor of group (CA vs. DY) and the within-subject factors of condition (A vs. V vs. AV) and channel (Fz vs. Cz vs. Oz). The dependent variable was the peak *r*-values. The main effect of group approached significance [*F*_(1, 30)_ = 2.999, *p* = 0.094, η^2^_*p*_ = 0.091]. There was a significant main effect of condition [*F*_(2, 60)_ = 6.675, *p* = 0.002, η^2^_*p*_ = 0.182]. Bonferroni corrected *post-hoc* analysis showed that this was driven by larger *r*-values in the A and AV conditions than in the visual condition (*p* = 0.024 and *p* = 0.016, respectively). The peak *r*-values in the A and AV conditions did not differ (*p* > 0.05). There was also a significant effect of channel [*F*_(2, 60)_ = 11.328, *p* = 0.001, η^2^_*p*_ = 0.274]. *Post-hoc* analysis revealed this to be driven by larger *r*-values at Cz than at both Fz and Oz (*p* < 0.001 and *p* = 0.01, respectively). A significant condition × channel interaction was also found [*F*_(4, 120)_ = 7.304, *p* < 0.001, η^2^_*p*_ = 0.196]. This would be expected, as stimulus representation should differ at each channel in different experimental conditions. The stimulus-response cross-correlation has a period of ~500 ms (see Figure 5). This suggests that it is dominated by delta band activity.

To investigate the *timing* of maximal response representation, we extracted the lags for which peak *r*-values occurred for each participant at each channel and in each condition (a subset of which is plotted in Figure 5). We then took the lags at each channel as the dependent variable in 3 separate ANOVAs, each with the between-subject factor of group and the within-subject factor of condition (A vs. V vs. AV). The ANOVA for Fz showed a main effect of condition [*F*_(2, 60)_ = 59.09, *p* < 0.001, η^2^_*p*_ = 0.653]. There was no significant effect of group, nor was there a significant group × condition interaction. Similar results were found for the Cz ANOVA [main effect of condition: *F*_(2, 60)_ = 33.808, *p* < 0.001, η^2^_*p*_ = 0.53]. However, given that the analysis of entrainment had shown that visual activity at Cz was not significantly entrained, we also carried out an exploratory Two-Way ANOVA for Cz omitting the visual condition, with factors of group (CA vs. DY) and condition (A vs. AV). Here we found a significant main effect of group [*F*_(1, 30)_ = 5.859, *p* = 0.022, η^2^_*p*_ = 0.163], paralleling the results found at Cz for the preferred phase of entrainment analysis. *Post-hoc* analysis of the group effect revealed that it was driven by controls having a *longer* peak-lag than the dyslexic group. Although the timing of peak stimulus representation (as identified by the peak lags) does not measure the same thing as preferred pre-stimulus phase, both are measures of the timing of the relevant oscillatory response activity. Indeed, the results of this peak-lag analysis mirror those of the preferred phase of delta entrainment analysis carried out above, as both analyses point to atypical timing of response entrainment and atypical response representation in participants with dyslexia. Both the strength of stimulus representation and response timing are likely to be crucial factors in phonological development.

Converging evidence for a potentially important role for the neural timing of auditory responses in phonological development and reading development was sought by exploring correlations between these two measures of the quality of stimulus representation and the behavioral measures. Peak *r*-values and peak lags at Cz in the three conditions were correlated with the various reading and phonology measures, partialling out age and IQ (see Table 5). A series of significant correlations were found, most notably in the Auditory condition, and the correlations were positive, suggesting that a stronger stimulus representation (higher peak *r*-value) and a longer peak lag were related to higher scores on the behavioral tasks. As shown in Table 5, peak *r-values* were significantly correlated with reading age, non-word reading and phoneme deletion in the Auditory condition, while peak lag was significantly correlated with reading standard score and reading age (The bottom panel of Figure 6 shows a scatter plot and regression line for the relationship between peak *r*-values at Cz in the auditory condition vs. performance in the phoneme deletion task) For the AV condition, peak *r*-values were significantly correlated with phonological awareness, while individual differences in peak lag were significantly correlated with reading age and RAN. As those with dyslexia showed *shorter* lags than controls, the more *“control-like”* the peak lag, the better the behavioral performance.

**Table 5:** **Partial correlations across all subjects controlling for age and IQ between reading and phonology measures and peak *r*-value and peak lag at Cz in the Auditory condition, Oz in the visual condition, and Cz for the audio-visual condition**.

**Measure**	***r*-value for A at Cz**	**Peak lag for A at Cz**	***r*-value for V at Oz**	**Peak lag for V at Oz**	***r*-value for AV at Cz**	**Peak lag for AV at Cz**
BAS(SS)	0.402^*^	0.409^*^	0.076	−0.003	0.182	0.308
BAS(AS)	0.401^*^	0.429^*^	0.079	0.008	0.171	0.31
Reading age	0.324^+^	0.388^*^	0.088	0.038	0.116	0.373^*^
TOWRE word reading	0.281	0.356^+^	0.068	0.07	−0.043	0.354^+^
TOWRE non-word reading	0.385^**^	0.356^+^	0.131	−0.087	0.033	0.241
RAN	−0.167	−0.322^+^	−0.111	−0.006	0.193	−0.467^**^
pSTM	−0.054	0.060	0.127	−0.133	−0.2	0.357^+^
Phoneme deletion	0.475^**^	0.229	0.094	0.064	0.466^*^	−0.107

** p < 0.01,

* p < 0.05,

+ p < 0.1.

Overall, the partial correlations suggest that the typically-developing children had stronger neural representations of the speech stimulus “ba,” and that the strongest representation occurred later in time compared to those with dyslexia. These results provide converging evidence for the importance of the *phase* of low frequency oscillations in stimulus encoding. The participants with dyslexia appear to be entraining to a sub-optimal phase, and this is reflected in both timing differences in their neural responses and also a difference in the quality of stimulus representation as measured by the correlograms.

To investigate potential effects of hemisphere on the strength of auditory stimulus representation and timing, we subjected the peak *r*-values and lags to separate 2 × 3 × 2 ANOVAs with a between-subject factor of group and within-subject factors of electrode pair (T3,T4 vs. T5,T6 vs. Tp7,Tp8) and hemisphere (left vs. right). The peak *r*-value ANOVA found no significant effects, suggesting that the strength of auditory stimulus representation does not differ by group in temporal regions. Furthermore, no hemispheric difference or interactions were found, suggesting that the strength of stimulus encoding is similar in both hemispheres. The lag ANOVA showed a main effect of Group [*F*_(1, 30)_ = 4.37, *p* = 0.045, η^2^_*p*_ = 0.127]. No other contrasts resulted in significant effects. This timing difference was again driven by children with dyslexia having a shorter peak lag than children in the control group.

### Phase resetting: effects of visual stimulation on auditory entrainment

**Figure 7:**
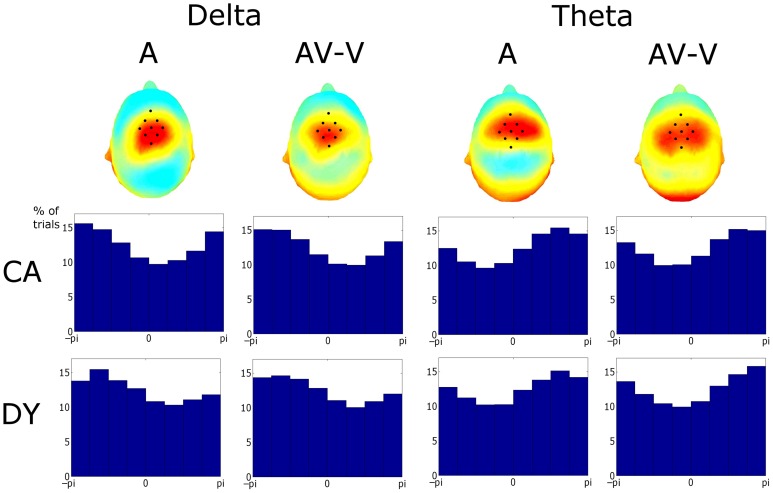
**Upper Panel:** Topographies of the ITC for phase at stimulus onset. The similarity of these topographies established a particular fronto-central region of interest coinciding with the area of strongest entrainment. **Lower Panel:** phase distributions for the conditions and frequency bands in the region of interest (ROI). Activity in the ROI showed significant entrainment, as tested using the Rayleigh statistic, in both frequency bands and for both response types and both groups. Tests on the preferred phase of entrainment showed that auditory delta phase differed between groups. Auditory theta phase differed between conditions and was thus affected by visual cues.

Following Power et al. (2012b), our final question was whether there would be group differences in the degree to which visual speech information would reset the phase of auditory oscillations so that they were optimally timed to encode the speech signal. Given behavioral data (e.g., Megnin-Viggars and Goswami, 2013), we expected that the dyslexic group might accrue greater benefit from visual phase-resetting than controls. Following Power et al. (2012b), Figure 7 shows ITC topographies for the A and (AV-V) conditions averaged across groups. The fronto-central distribution in both conditions is indicative of entrainment in auditory cortical areas. Figure 7 also shows the phase distributions for delta and theta for the pooled activity in the ROI for both groups and conditions. Rayleigh tests revealed significant entrainment in both conditions at both frequencies (A_δ__DY: *Z* = 403.43, *p* << 0.001; AV-V_δ__DY: *Z* = 265.87, *p* << 0.001; A_θ__DY: *Z* = 441.16, *p* << 0.001; AV-V_θ__DY: *Z* = 376.69, *p* << 0.001; A_δ__CA: *Z* = 1247.30, *p* << 0.001; AV-V_δ__CA: *Z* = 684.82, *p* << 0.001; A_θ__CA: *Z* = 1220.5, *p* << 0.001; AV-V_θ__CA: *Z* = 704.96, *p* << 0.001). This would be expected given the way in which the ROI was determined.

To assess possible group differences in the effects of visual speech cues on the auditory oscillations, we first investigated whether the level of auditory entrainment (inter-trial coherence, ITC) was affected by the visual cues. The ITC values were submitted to two 2 × 2 ANOVAs (one each for delta and theta), with the between-subject factor of group (CA vs. DYS) and within-subject factor of condition [A vs. (AV-V)]. The ANOVAs showed no main effect of group nor condition in either frequency band (all *p*'s > 0.05), suggesting that the strength of auditory phase locking in both bands was similar whether visual cues were present or not. There was also no significant group × condition interaction in either band (both *p*'s > 0.05).

Mirroring the previous ITC analysis, we next carried out a similar 2 × 2 ANOVA for each frequency band taking the overall response power as the dependent variable. In the delta band, we found no main effect of group, but a significant group × condition interaction [*F*_(1, 30)_ = 5.809, *p* = 0.022, η^2^_*p*_ = 0.162]. *Post-hoc* inspection revealed that the interaction was driven by higher delta power in the A than in (AV-V) for the control children only. There was no difference in power between the two conditions for those with dyslexia. The theta band ANOVA showed similar results, with no main effect of group, but a significant group × condition interaction [*F*_(1, 30)_ = 5.048, *p* = 0.032, η^2^_*p*_ = 0.144]. *Post-hoc* inspection revealed that this was again driven by higher power in A than AV-V for the control children only.

Taking these results together, typically-developing children showed a significant difference between auditory oscillatory activity to auditory stimuli alone (A) and auditory oscillatory activity when visual cues were present (AV-V), in both delta and theta power. The children with dyslexia did not. This may indicate that auditory cortex in typical development does not have to work as “hard” to process speech stimuli when they are presented multimodally. The results also show that the consistency of auditory phase is not affected for either group by whether stimuli are only auditory or whether they are audio-visual.

### Preferred pre-stimulus phase

To assess whether the information from visual speech affected the phase of auditory entrainment similarly for each group, we tested for preferred phase differences in each frequency band using two 2-way circular ANOVAs (HK ANOVA as before). Each ANOVA had group as the between-subjects factor (CA vs. DY) and condition [A vs. (AV-V)] as the within-subjects factor. In the delta band ANOVA there was a significant main effect of group [*F*_(1, 63)_ = 11.06, *p* = 0.0015]. This mirrors the differences in preferred phase found by group at Cz for the auditory condition. There was no significant effect of condition (*p* > 0.05) and there was no significant interaction (*p* > 0.05). In the theta band ANOVA we found a significant main effect of condition [*F*_(1, 63)_ = 7.97, *p* = 0.0065] but no significant main effect of group (*p* > 0.05) and no significant interaction (*p* > 0.05). This suggests that for theta the preferred auditory phase in the ROI is altered by audio-visual information. The absence of a significant interaction with group in both ANOVAs suggests that the preferred phase of entrainment in both frequency bands is similarly affected by visual speech information in both groups, with no phase alteration in the delta band but a significant phase alteration in the theta band. Nevertheless, the preferred delta phase at which auditory responses entrain is different between the groups. Overall these data suggest that for theta band entrainment, which by hypothesis is primary in syllable-level processing (Poeppel, 2003), accompanying visual speech information does alter the preferred phase of entrainment, for both groups. Therefore, accompanying visual information results in a more optimal theta phase than when auditory information is presented alone, and both groups are *similarly* affected by visual speech information. There is no evidence for enhanced use of visual speech information by participants with dyslexia. In contrast, group differences in the preferred delta band phase persist in spite of the visual speech information. This suggests that *sub-optimal phase of entrainment* still occurs in the AV condition for participants with dyslexia.

## Discussion

Here we compared neuronal oscillatory entrainment in children with and without dyslexia in the delta and theta bands to a rhythmic speech stimulus, the syllable “ba” repeated at a 2 Hz (delta) rate. The speech stimulus was either presented in the auditory modality only, the visual modality only, or audio-visually (AV). On the basis of the temporal sampling framework for developmental dyslexia (TSF, Goswami, 2011), we predicted group differences in entrainment in the auditory modality. Given the prior literature on oscillatory entrainment in dyslexia (adult studies, Hämäläinen et al., 2012b; Soltesz et al., 2013), delta band oscillations seemed the most likely to reveal group differences in the current study. On the basis of previous behavioral studies of entrainment (tapping measures) with adults and children, we again expected group differences in the delta band (Thomson et al., 2006; Thomson and Goswami, 2008). Finally, on the basis of recent studies of behavioral entrainment in adults with dyslexia to rhythmic speech, we predicted possible group differences in preferred phase alignment (Leong and Goswami, 2013).

Here the data in the auditory entrainment condition showed no difference in phase consistency (ITC) over trials between the groups, and no difference in response power between groups. However, significant differences were indeed found in the *timing* of auditory stimulus encoding. Timing differences were revealed both by a significant group difference in the *preferred phase* of neuronal entrainment in the delta band, in both the auditory and AV conditions, and by the timing of maximal stimulus encoding as measured by cross-correlating the stimulus envelope with the neural response. The cross-correlation approach revealed a significant group difference in peak lag value, with typically-developing children showing later peak lags than children with dyslexia. There was also a trend toward higher *r*-values in controls, indicating better stimulus envelope representation. Regarding laterality, we found no differences in peak *r*-values by group or hemisphere, although longer peak lags were found in both hemispheres in controls. This is discussed further below. Individual differences in both the preferred delta phase measure and the cross-correlation measures were significantly correlated with behavioral measures of reading and phonology (Tables 4, 5). The preferred delta phase measure in the auditory condition showed a particularly consistent set of relations, with significant correlations for all the measures of reading and the phoneme deletion measure.

The suboptimal phase of encoding demonstrated for the participants with dyslexia in the delta band is likely to have significant consequences for the quality of their phonological representations. According to MTRMs of speech encoding (e.g., Luo and Poeppel, 2007; Ghitza and Greenberg, 2009), speech input is encoded most efficiently by the brain when endogenous cortical neuronal oscillations phase-align with temporal modulations (amplitude or frequency modulations) in the input signal, so that maximal neuronal responses occur at the most informative points. If the phase of peak neural responding is consistently misaligned with the modulation peaks in the input, then the signal will be encoded in suboptimal fashion. This will result in differently-specified phonological representations for words in the mental lexicon. The cross-correlation analyses in the current study (which cross-correlated the neural response with the stimulus envelope) provided congruent evidence for significantly different neural timing (peak lag measure) and lower quality neuronal representation of the speech envelope for “ba” (peak *r-value* measure) by the children with dyslexia. These delta band findings suggest that the highest level in the amplitude modulation hierarchy, the delta band, which carries information about prosodic structure, is encoded less efficiently by the dyslexic brain. This would have cascading effects for the encoding of the other levels of phonological structure that are nested within the delta band, including syllable-level (theta band) AM information and phoneme-level (gamma band) AM information. The difference in preferred phase in the current study was 12.8 ms between groups (0.1613 radians at 2 Hz). Acoustic changes in this timescale would be in the gamma range, suggesting that the consistent timing difference in preferred delta phase shown by our participants with dyslexia would have cascading consequences for the optimal encoding of gamma-rate or phonetic information. Some of these faster gamma transitions would be occurring in a sub-optimal temporal window, contributing to the impairments in phonological encoding found at every grain size (prosodic, syllabic, onset-rhyme and phonemic) in developmental dyslexia (Snowling et al., 2000; Ziegler and Goswami, 2005; Goswami et al., 2013).

Hemispheric differences were not found in the cross-correlation measures, in contrast to an earlier study by Abrams et al. (2009). Abrams and colleagues employed three stimulus types: clear speech, conversational speech and compressed speech. A right hemisphere dominance in stimulus encoding (peak *r-value*) was found for the clear and conversational speech in good and poor readers, whereas for time-compressed speech (a more challenging listening condition), the right hemisphere dominance was only found for good readers. Encoding was represented symmetrically across hemispheres for the poorer readers. Although we found no hemispheric differences in encoding, there are some possible reasons for the differing results. Crucially, Abrams et al. (2009) employed a paradigm in which stimuli were presented to the right ear only. Subjects were instructed to ignore the sentences and to attend to a movie whose soundtrack was presented to the left ear. It has been shown that spatial attention in a speech environment suppresses the neural representation of the ignored stimulus (Horton et al., 2013). It has also been reported that stronger speech suppression takes place in the left hemisphere than in the right under such conditions Power et al. (2012a). The interplay between attention and hemispheric bias is not addressed in Abrams et al. (2009), and it is possible that attentional influence may contribute to the reported right hemisphere bias. For example, the fact that the stimulus is being actively ignored may suppress stimulus representations in the left hemisphere more than in the right. The fact that our stimuli are presented rhythmically, and thus timing is entirely predictable from syllable to syllable, is a further important difference with Abrams et al. (2009), where the speech stimulus envelope was not periodic. Indeed, the fact that the Group × Hemisphere interaction was only seen in their compressed speech condition suggests that the hemispheric interaction effect may be only apparent when the auditory system attempts to entrain to a taxing stimulus whose envelope is variable.

Abrams et al. (2009) also found a significant group × hemisphere interaction for peak lags in all three speech conditions. Poor readers had earlier *r*-value peaks in the left hemisphere and later peaks in the right hemisphere. It is of note that our results mirror the left hemisphere timing findings of Abrams et al. (2009). The lack of Group × Hemisphere interactions in our study may be due in part to the predictable nature of our stimuli. If the right hemisphere does preferentially encode low frequency activity, as hypothesized by Poeppel (2003), and if this right hemisphere encoding network is the primary impairment in dyslexia, as hypothesized by our group (Goswami, 2011), then we can argue that in a case where the right hemisphere network's capacity to follow low frequency fluctuations is not heavily taxed (as with a rhythmic and predictable stimulus), hemisphere differences may not be found. However, when difficulty increases (such as with non-periodic speech), the unaffected right hemisphere of control participants can facilitate processing, resulting in a decreased peak lag. In contrast, the impaired right hemisphere network of participants with dyslexia will struggle to cope, and so the peak lag increases. Taken together, the results of both studies converge in showing impaired processing of low frequency information by poor readers, both in terms of strength of stimulus representation and response timing. Further research is required, however, to tease apart the delicate contributions of attention and stimulus parameters.

Indeed, a recent study exploring how new acoustic representations are learned by the adult brain (Luo et al., 2013) has shown that neuronal phase patterns in *low-frequency oscillatory responses* below 8 Hz (i.e., in the delta and theta bands) are critical to the learning process. Distinguishably-different low-frequency oscillatory phase patterns were shown by Luo and colleagues to form gradually over learning time, thereby differentiating novel noise patterns as individual auditory objects for successful learners. If a similar learning mechanism underpins the learning of the acoustic patterns which are words, then the phase differences in dyslexia in the delta band revealed here would have serious consequences for the quality of the phonological representations of word forms developed by affected children. Oscillatory phase patterns may be more important than oscillation amplitude in terms of informational encoding. Ng et al. (2013) used natural animal sounds to investigate the encoding of acoustic stimuli in macaque auditory cortex, examining neural firing directly by recording local field potentials inside the brain. Ng and colleagues showed that stimulus-selective firing patterns imprinted on the *phase* rather than the *amplitude* of slow oscillations (<8 Hz), with phase patterns rather than oscillation power carrying discriminative information. A comparable result was reported for human EEG to the same naturalistic stimuli, and Ng and colleagues noted that these naturalistic stimuli could be discriminated on the basis of their phase patterns *without* any increases in oscillatory power. The emerging importance of phase suggests that the brain capitalizes on both power (firing rate) and phase (the timing of firing) when encoding and developing neuronal representations for a complex stimulus like human speech. Therefore, the neural timing differences revealed in the current study could carry important implications for the *quality* of phonological encoding. Note that earlier ASSR studies measuring differences in response *power* between adult participants with and without dyslexia did not measure phase consistency across trials (Lehongre et al., 2011; Poelmans et al., 2012). The identified difference in grand averaged power in those studies may hence be due to inconsistent phase alignment across trials. Both firing rates and phase patterns tend to be sensitive to the same acoustic features (Ng et al., 2013). Hamalainen et al. did investigate both phase and power in their ASSR study, and in their MEG study the group differences between participants with dyslexia and controls at 2 Hz were caused by differential phase consistency and not by differential response power. Note further that in the non-speech study reported by Soltesz et al. (2013), phase *was* examined, and those with dyslexia did show an earlier preferred phase in the 2 Hz entrainment condition compared to the control group; however, this effect was not significant. Nevertheless, it is important to note that none of these earlier dyslexic studies used the speech signal as input.

Contrary to prediction, we did not find any significant differences in *visual entrainment* between children with dyslexia and control children. As noted earlier, differences between dyslexic and control children have been found in visual attention shifting tasks (e.g., Facoetti et al., 2010) and in visual attention span measures (e.g., Lallier and Valdois, 2012), while adults with dyslexia have been reported to show *superior* perception of and memory for low-frequency visual features in natural scenes (Schneps et al., 2012). Our task explored the neural processing of natural dynamic visual cues to speech perception, which incorporate both low-frequency (e.g., jaw movement) and high-frequency (e.g., lip shape) visuo-spatial information, and by hypothesis should be directly related to the quality of phonological encoding. However, in the visual alone condition, dyslexics and controls showed equivalent entrainment strength and equivalent preferred phase, while in the AV condition the dyslexic group again showed an earlier preferred phase in the delta band compared to control participants, mirroring the findings for the auditory alone condition. When we explored how visual speech information affected the phase of auditory entrainment, we found that in the theta band visual information did alter preferred auditory phase, but to the same extent for both groups. Visual speech information is thought to reset auditory theta phase to the optimal alignment for processing upcoming speech (Schroeder and Lakatos, 2009). The only significant group difference was again in the delta band. As in the auditory alone condition, when computed for (AV–V), preferred phase of entrainment was significantly earlier for the dyslexic group. Hence despite the accompanying visual information, in the AV condition the participants with dyslexia were again entraining to a suboptimal phase. As previously, this suggests that the slower delta oscillations are not providing the dyslexic brain with an efficient temporal reference frame for auditory information encoding. In the theta band, by contrast, both groups showed efficient phase resetting of auditory oscillatory activity by congruent visual information.

In fact, given the earlier study by Power et al. (2012b) using the current paradigm, which reported a significant relationship between theta power and reading development in typically-developing children, the absence of significant group differences in theta band entrainment in the current study is somewhat surprising. Theta entrainment is thought to be central to speech processing on multi-time resolution models (syllable-level entrainment, e.g., Luo and Poeppel, 2007). However, our failure to find group differences in theta power or phase could be task-related. The participants were required to process a delta-rate rhythm (2 Hz), and to detect violations of that rhythm, and thus task demands did not focus on theta entrainment or phase. If stimuli had been delivered instead at a rhythmic rate within the theta band (e.g., 5 Hz), group differences in theta activity may have emerged. Nevertheless, the current violation detection task is likely to be more informative than the passive entrainment tasks used in prior studies with adult dyslexics (Lehongre et al., 2011; Hämäläinen et al., 2012b; Poelmans et al., 2012). With a passive listening paradigm it is impossible to quantify how the different groups are approaching the task, for example whether those with dyslexia and controls are using similar processing strategies. Furthermore, prior oscillatory studies suggest that when a stimulus is continuous (rather than rhythmic, as utilized here), the brain uses a continuous mode of processing, which maximizes gamma activity (e.g., Schroeder and Lakatos, 2009). Hence the gamma findings in prior studies using non-speech and continuous stimuli (Lehongre et al., 2011; Poelmans et al., 2012), indicating that gamma power was significantly lower in the dyslexic group when processing AM noise, could reflect task demands rather than stimulus-specific processing differences between participants with dyslexia and controls.

In conclusion, this study provides direct neural evidence for the “phonological representations” hypothesis of developmental dyslexia, according to which the neural representations underpinning word recognition in children with dyslexia are impaired or atypical in their phonological characteristics. The current study suggests that one mechanism contributing to atypical development of the dyslexic mental lexicon is auditory oscillatory entrainment to speech at a different preferred phase of the delta band, which consequentially affects the quality of the information encoded at all phonological levels including the phonemic level. Concurrent visual speech information as in natural listening conditions is not sufficient to ameliorate this difference in preferred auditory phase, as shown by the AV condition in the current study. Nevertheless, converging evidence is required regarding the developmental salience of delta band information for developing high-quality phonological representations, ideally investigating the entrainment to, and encoding of, auditory and speech stimuli in the dyslexic brain under various task demands.

### Conflict of interest statement

The authors declare that the research was conducted in the absence of any commercial or financial relationships that could be construed as a potential conflict of interest.
